# The occipital face area is causally involved in identity-related visual-semantic associations

**DOI:** 10.1007/s00429-020-02068-9

**Published:** 2020-04-27

**Authors:** Charlotta Marina Eick, Gyula Kovács, Sophie-Marie Rostalski, Lisa Röhrig, Géza Gergely Ambrus

**Affiliations:** Biological Psychology and Cognitive Neurosciences, Institute of Psychology, Friedrich Schiller University Jena, Leutragraben 1, 07743 Jena, Germany

**Keywords:** Semantic face processing, Transcranial magnetic stimulation, Occipital face area, Face processing network, Semantic information, Recognition

## Abstract

Faces are processed in a network of areas within regions of the ventral visual stream. However, familiar faces typically are characterized by additional associated information, such as episodic memories or semantic biographical information as well. The acquisition of such non-sensory, identity-specific knowledge plays a crucial role in our ability to recognize and identify someone we know. The occipital face area (OFA), an early part of the core face-processing network, is recently found to be involved in the formation of identity-specific memory traces but it is currently unclear if this role is limited to unimodal visual information. The current experiments used transcranial magnetic stimulation (TMS) to test whether the OFA is involved in the association of a face with identity-specific semantic information, such as the name or job title of a person. We applied an identity-learning task where unfamiliar faces were presented together with a name and a job title in the first encoding phase. Simultaneously, TMS pulses were applied either to the left or right OFA or to Cz, as a control. In the subsequent retrieval phase, the previously seen faces were presented either with two names or with two job titles and the task of the participants was to select the semantic information previously learned. We found that the stimulation of the right or left OFA reduced subsequent retrieval performance for the face-associated job titles. This suggests a causal role of the OFA in the association of faces and related semantic information. Furthermore, in contrast to prior findings, we did not observe hemispherical differences of the TMS intervention, suggesting a similar role of the left and right OFAs in the formation of the visual-semantic associations. Our results suggest the necessity to reconsider the hierarchical face-perception models and support the distributed and recurrent models.

## Introduction

Faces are processed in regions along the ventral object vision pathway (Ungerleider and Haxby 1994). However, face processing deviates from object or body processing, as faces also carry conceptual knowledge of the person (Lambon Ralph et al. 2009; Pitcher et al. 2009). The recognition of a person involves the process of identifying the face as well as learning and recalling the associated person-related knowledge, such as a name, associated and relevant places or the occupation a person. The first influential cognitive model of face recognition by Bruce and Young (1986) considered the two functional components of ‘recognizing a face’ and that of ‘encoding the relevant identity-specific semantic information’ to be inseparable. Addressing the criticism that these models assumed the existence of a single “face recognition unit” and were not based on neurophysiological data, Haxby et al. (2000) further developed the model into a neuronally motivated, two-stage network model. The core of this model, comprising the fusiform (FFA) and occipital face areas (OFA) as well as the superior temporal sulcus (STS), is involved in the early visual processing of faces. The extended system, on the other hand, including the anterior temporal pole (ATL), the amygdala, parietal as well as frontal areas (Gobbini and Haxby 2007; Rapcsak 2019), is active in higher-level cognitive processing such as semantic knowledge, emotional and motivational evaluation as well as personality traits (for a review see Ramon and Gobbini 2017).

Previously, the face-processing network was seen as a strictly hierarchical system organized in a feedforward manner (Haxby et al. 2000; Ishai 2008; Pitcher et al. 2011; Zhen et al. 2013). In these models, it was assumed that information flows from the early visual cortex toward OFA, where face detection occurs and then on to FFA for individual face recognition and to the ATL, amygdala and STS, where more complex processing, such as the identification of individual persons takes place.

In the following years, all the above-mentioned areas were studied in detail for their particular functions, often by using transcranial magnetic stimulation (TMS) where applicable (Ambrus et al. 2017a, 2019; Kadosh et al. 2011; Koivisto et al. 2017; Ranieri et al. 2015).

The highest-level unimodal area, involved in face perception, prior to memory and emotional processing is presumed to be the ATL, also called the anterior face patch (aFP; Harry et al. 2016; Rajimehr et al. 2009). Several studies found that the ATL is of importance in the identification of a person (Heide et al. 2013; Tsao et al. 2008; Tsukiura et al. 2010). Accordingly, recent TMS studies showed that stimulating the right ATL affects the feeling of familiarity for famous faces (Ranieri et al. 2015). It has been suggested that the ATL connects the visual representation of a face to the corresponding semantic knowledge one possesses about the given person (Chiou and Lambon Ralph 2018; Rice et al. 2018; Tsukiura et al. 2010). Also, hierarchically lower face-processing areas, such as the FFA, were associated with face recognition processes in the past. For instance, the FFA shows habituation to different images of the same individuals (Gauthier et al. 2000), while the OFA does not (Rotshtein et al. 2005). More recently, Axelrod and Yovel (2015) have found significant decoding of famous faces in the FFA, using multivariate pattern analysis techniques as well. These results altogether suggest that individual faces are already processed differentially at the level of the FFA and newer theories assume the FFA to be an intermediate stage of face processing. Until recently, it was believed that this is not the case for the OFA, which area is typically considered as an early, structural face-processing stage (Pitcher et al. 2007; but see Rossion (2014) for a different conclusion). However, current results suggest that the OFA is also involved in processes related to face recognition. For example, if the right OFA (rOFA) is lesioned, patients show impaired face discrimination capacities (Dricot et al. 2008; Schiltz et al. 2006). Furthermore, repetitive double-pulse TMS of the rOFA disrupts the discrimination of face parts (Pitcher et al. 2007, 2008) as well as the integration of identity and expression (Kadosh et al. 2011). Zhen et al. (2013) also argue that the OFA is involved in face identification and has a pivotal role in the face-processing network. In accordance, recent evidence suggests that the rOFA is important to identity-specific processing and to the learning of novel faces, as TMS over the rOFA (1) eliminates the effects of training in a face-matching task (Ambrus et al. 2017a), (2) reduces the observed face priming effects (Ambrus et al. 2017b) and (3) impairs discrimination of unfamiliar faces (Pitcher et al. 2008, 2009). Moreover, the participation of the OFA in processing identity-specific information is not limited to visual facial information. Amado et al. (2018) showed that the recognition of a familiar face is facilitated by the prior presentation of its name and this cross-domain priming effect is related to a reduced BOLD signal in bilateral FFA and OFA. Later, it has been demonstrated that the TMS of rOFA reduces these cross-domain priming effects, showing that the area plays a causal role in the association of semantic and visual information (Ambrus et al. 2019).

Altogether, these results imply that the OFA is more than a simple low-level, structural encoding area (Haxby et al. 2000) and that it is important to identity-specific visual and semantic processing. Traditionally, assessing personal information of a face is described as a serial process, starting with the activation of face-related information and then assessing person- and finally name-related semantic information (Tsukiura et al. 2010). Additionally, sub-networks that correspond to the recognition of an individual’s face and the retrieval of semantic knowledge are considered to be relatively independent processes (Zhen et al. 2013). Besides foundational features of the network that suggest a clearly serial processing, such as the continuously growing receptive field size along the hierarchy (Grill-Spector et al. 2017), a strictly hierarchical model of visual processing is increasingly put into question (Atkinson and Adolphs 2011). Indeed, a more complex pattern of connectivity among areas of the face network has been uncovered recently. For example, the OFA is in close exchange with the FFA via feedback connections (Gschwind et al. 2012; Jiang et al. 2011; Rossion et al. 2003). In line with this result, Kadosh et al. (2011) suggest that the OFA receives re-entrant feedback information at a mid-latency processing stage, around 170 ms. Hence, it seems that the neural response to familiar faces in the ventral occipito-temporal cortex (including the inferior occipital gyrus) reflects feedback from other face-sensitive areas as well (Gobbini et al. 2004). Furthermore, the existence of functional top-down connections from the ATL to the ventral temporal cortex has been demonstrated in object recognition (Chiou and Lambon Ralph 2016) and robust structural connectivity is reported between ATL and FFA as well as OFA (Pyles et al. 2013). Particularly, when top-down guided processes are involved, such as mnemonic functions, attention or predictions, or in this case getting to know an originally unfamiliar face via various resources, the reverse hierarchy of perceptual processes is proposed to gain more weight (Ahissar and Hochstein 2004). In summary, it seems that the face-processing neural network is far more interactive than originally thought and it has a strong interplay between the involved neural regions which may also depend on non-visual parameters, such as the task (Atkinson and Adolphs 2011; Barragan-Jason et al. 2013; Kadipasaoglu et al. 2017).

The current experiments further investigate the role of bilateral OFA in the acquisition of identity-specific semantic information. To this end, we adopted a learning paradigm where basic semantic information (the name and job of a person) was associated with a face. Importantly, TMS was applied to either the left or right OFA during the encoding phase and the participants’ performance was measured in a subsequent retrieval phase.

Face processing is a bilateral process despite its clear right hemispherical (RH) dominance (Haxby et al. 2000; Kanwisher et al. 1997). This RH dominance is evident in larger activated areas and larger face-selective event-related potential components on the right as compared to the left side (Bentin et al. 1996; Bukowski et al. 2013). This lateralization holds for faces in general (Gao et al. 2018; Rossion et al. 2012) as well as specifically for familiar face processing (Wiese et al. 2019; Wuttke and Schweinberger 2019). Interestingly, the lateralization is more evident, and it is connected to more specific functions at higher processing steps. For example, the right ATL is dominant for the processing of familiar faces, while the left ATL is more involved in the encoding of familiar names (Chiou and Lambon Ralph 2018; Ranieri et al. 2015; Rice et al. 2018). Therefore, to study the differential role of the two hemispheres in the acquisition of identity-specific semantic information, we stimulated the OFA of both hemispheres in separate experimental strands.

## Methods

### Participants

Forty-five healthy participants with no prior neurological or psychological conditions were included in the study (mean age (± SD) was 23.6 (4.2) of which 20 male). Twenty-two were stimulated over the left, the other 23 over the right OFA. One participant of the right hemisphere-stimulated group, however, performed below chance level and was excluded from the analysis. All participants were German native speakers, had normal or corrected to normal vision and were right-handed. No metal implants or medication with neurological impacts was reported. Written consent of all participants was obtained prior to the experiment, and they received either monetary compensation or partial course-credits. The guidelines of the Declaration of Helsinki were met and the study was approved by the ethics committee of the University of Jena.

### Localization of OFA

**Fig. 1:**
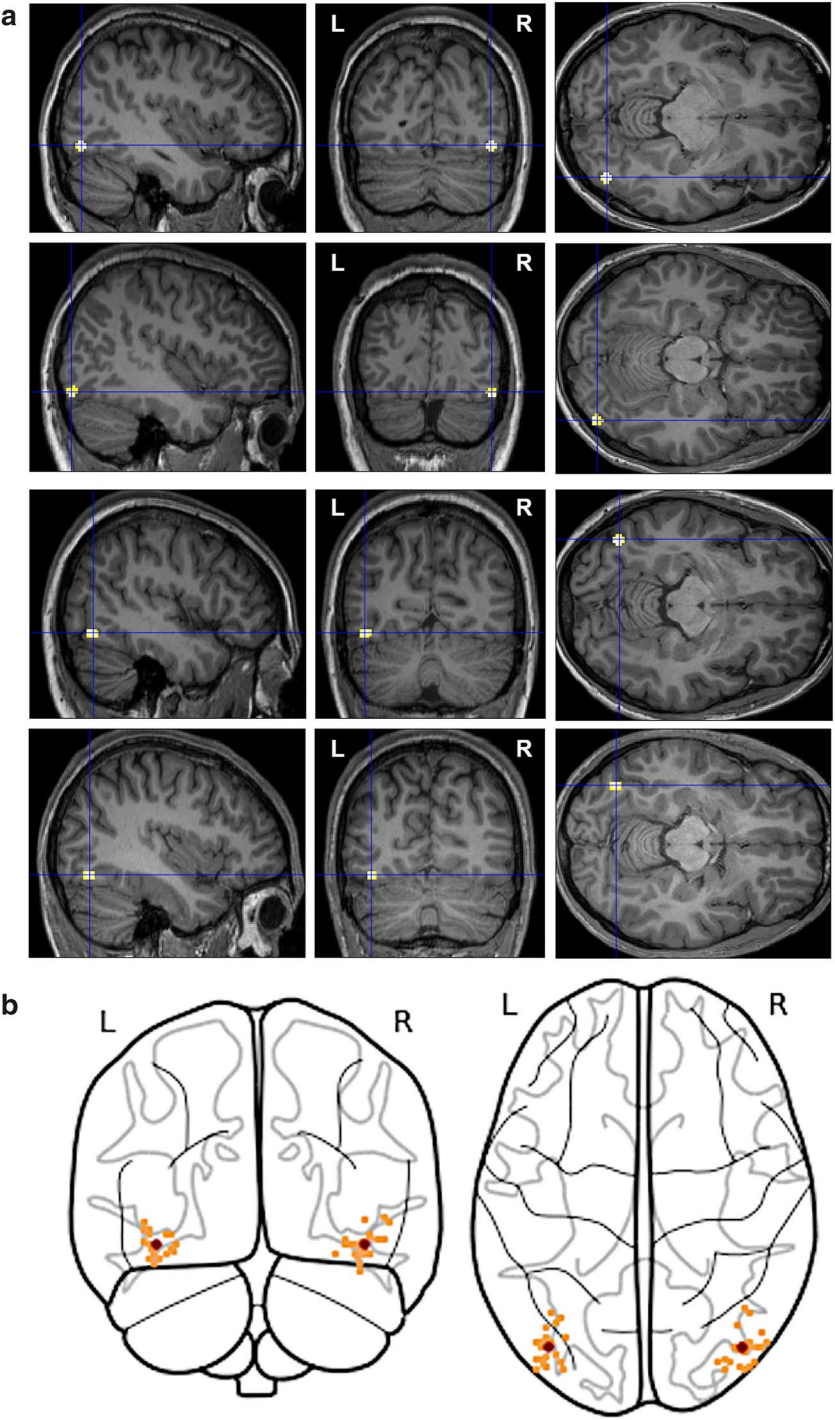
The position of the transcranial magnetic stimulation over the right and left occipital face area (**a**) in four representative individual participants on their native structural brain images (*p* < 0.05_FWE_) and (**b**) in an overview of all used coordinates (orange dots) as well as the mean for the left and right hemispheres (red dots; all figures plotted using MNI coordinates; glass brain, Python nilearn; Abraham et al. 2014)

To ensure the accurate position of the stimulation of the target area, the individual OFA was localized via functional neuroimaging measurements in every participant. Scanning was performed by a 3T magnetic resonance imaging (MRI) scanner (Siemens MAGNETOM Prisma fit, Erlangen, Germany) at the Institute for Diagnostic and Interventional Radiology, University of Jena. Structural T1-weighted images were obtained (MP-RAGE; TR = 2300 ms; TE = 3.03 ms; isotropic voxel size = 1 mm^3^), while for the functional MRI images, a Siemens 20-channel phased-array head coil and a gradient-echo, T2-weighted EPI sequence was used (35 slices; 10° tilted relative to axial; TR = 2000 ms; TE = 30 ms; flip angle = 90°; matrix array = 64 × 64; isotropic voxel size = 3 mm^3^).The functional scanning consisted of five blocks, each containing three 20 s series of colored images of faces of celebrities and unfamiliar persons, objects and Fourier-randomized noise (Dakin et al. 2002) patterns. The resting period between the blocks was 20 s and the stimulus presentation frequency was 4 Hz (230 ms exposition time with a 20 ms inter-stimulus interval), presented in Matlab (MathWorks, Version R2013). This stimulation frequency was found to elicit the highest selectivity in regions selective to bodies, faces, and places (Stigliani et al. 2015). Analysis, including pre-processing and statistics, was done using SPM12 (Wellcome Department of Imaging Neuroscience, London, UK, Version 12) in Matlab (MathWorks, Version R2018; for a detailed description of the pre-processing see Cziraki et al. 2010) to estimate the location of bilateral OFA (chosen as the local maximum from the *t*-maps with a threshold of *p* < 0.05_FWE_ or *p* < 0.0001_uncorrected_), by contrasting face-evoked vs. noise and object-evoked activations. The coordinates of the OFA were transferred from the MNI to the TAL system by GingerAle (Version 2.3). The average (± SE) MNI coordinates were 42 (1.1), − 79 (1.4), − 12 (1) and − 39 (0.8), − 78 (1.5), − 13 (1) for the right and left OFA, respectively (Fig. 1).

### Stimuli

100 grayscale portraits of unfamiliar, Caucasian identities (ID; half male) were selected from an in-house database. The face expressions were neutral or positive and the pictures were cropped to a size of 138 × 178 pixels (BenQ LED display, 1680 × 1050 pixel resolution and 60 cm viewing distance). For additional semantic information, 50 job titles and 50 German female and male forenames were collected. The names and job titles were read out by a computer-generated female voice via Microsoft Hedda Desktop (Version 11.0) and pre-recorded using an in-house Python script. The audio was presented via a commercially available loudspeaker. During the experiment, each face was allocated to one forename and job title randomly.

### Experimental procedure

**Fig. 2:**
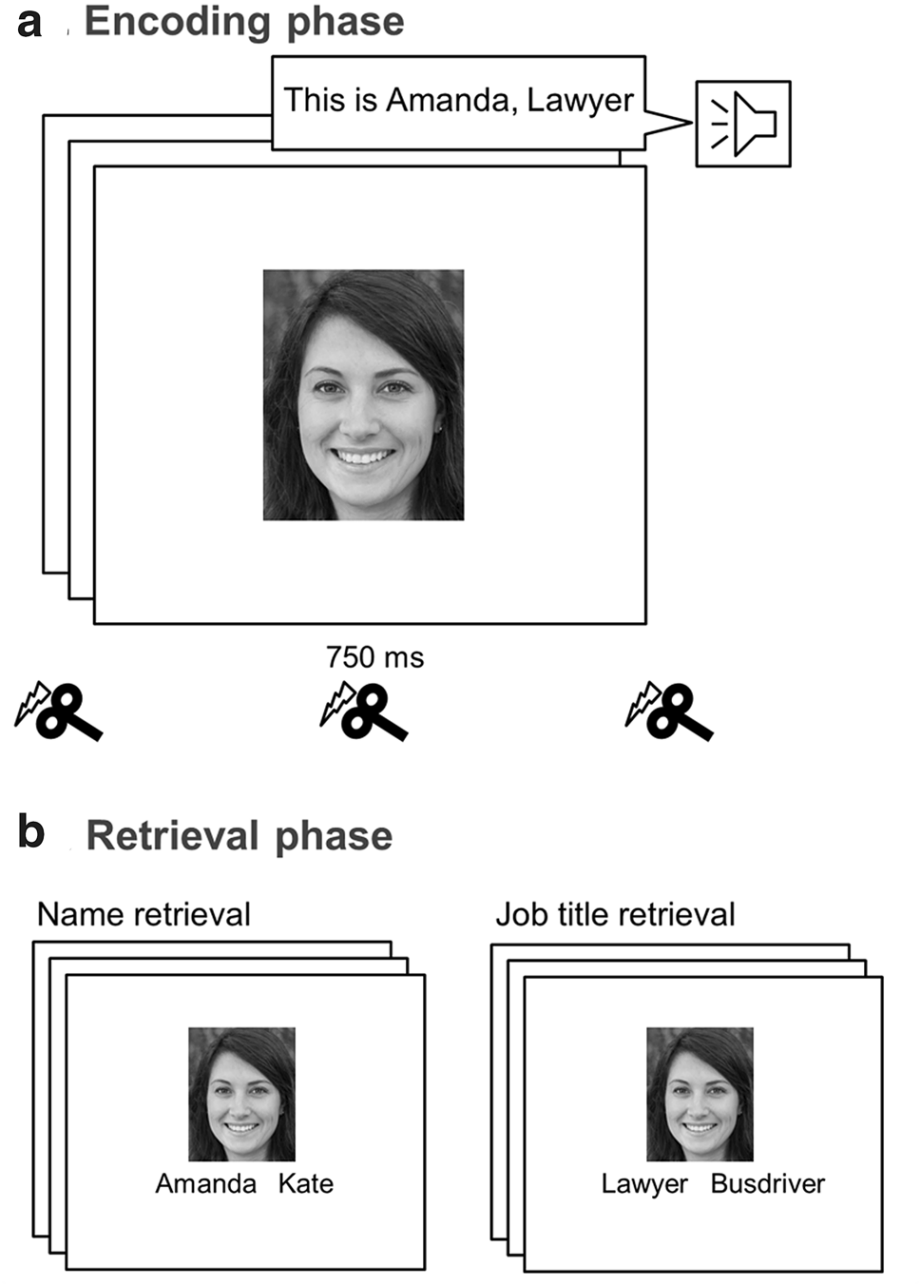
During the encoding phase, (**a**) 20 unfamiliar faces were presented twice for 750 ms with simultaneous acoustically presented semantic information, a name and a job title. Transcranial magnetic stimulation was delivered 100 ms prior, concurrent with and 100 ms after image onset over OFA or control. During the retrieval phase, (**b**) the previously seen faces were presented along with the associated name or job title together with a randomly selected gender-matching forename or job title. The participants’ task was to press a button signaling the correct name/job title. The depicted face image was artificially generated by Generative Adversarial Networks (www.thispersondoesnotexist.com)

The paradigm contained two phases. In the encoding phase (Fig. 2a), 1 of 20 randomly selected faces was presented for 750 ms on a gray background and simultaneously a sentence with 1 of 20 names and 20 job titles (duration 1000 ms) was presented acoustically, e.g., ‘This is Amanda, Lawyer’ (‘Sie ist Amanda, Anwältin’; German job title was gendered to match the male/female name). During this phase, TMS was applied at image onset either over OFA or Cz as control. The interstimulus interval (between the offset of the acoustic semantic information and the onset of the subsequent face) was 2000 ms. Each ID was presented twice, leading to 40 stimulus presentations during the encoding phase. There was no task for the participants except to remember the face and the associated name and job title. In the subsequent retrieval phase (Fig. 2b), the 20 trained faces were presented either with two names (the previously associated and a randomly selected gender-matching forename) or with two job titles (the previously associated and another randomly generated one) with equal probability in a random fashion (exposition time until button press with an interstimulus interval of 300 ms). Names and job titles were presented under the face images next to each other. The position (left or right) of the correct name or job title was randomized across trials (Fig. 2b). The task of the participants was to indicate the correct name/job title by pressing the left or right arrow key. Each of the 20 IDs was presented twice with forenames and twice with job titles, leading to 80 stimulus presentations during the retrieval phase.

The experiment was performed twice for each participant (with another set of 20 different face images, names and job titles each time), once with OFA and once with control TMS stimulation, with a separation of at least 4 days between the two sessions. The order of the OFA/control stimulation was randomized and counterbalanced across participants. A short training with four famous celebrities, not presented during the main experiment (Angela Merkel, Kim Kardashian, Silvester Stallone and Donald Trump), was performed by each participant prior to the experiment to ensure they understood the task (total duration was 15 min).

### Transcranial magnetic stimulation

Online stimulation was carried out with a PowerMag 100 Research Stimulator (MES Forschungssysteme GmbH). Stimulation intensity of 65% maximal stimulator output was chosen leading to an inhibitory effect of the neurons (Silvanto et al. 2017; Silvanto and Cattaneo 2017). Triple pulses were given at 100 ms prior, concurrent with and 100 ms after image onset during the encoding phase of the experiment. To enhance stimulation accuracy, the neuronavigation system PowerMag View (MES Medizintechnik GmbH) was applied to focus the center of the stimulation cone on the OFA or the control area, Cz. A chin rest stabilized the participants’ head and the handle of the stimulation coil pointed backward.

### Statistical analysis

The performance accuracy of correctly chosen names as well as job titles was analyzed.

To evaluate if TMS over the OFA influenced job title or name retrieval performance, a mixed ANOVA (between-subject factor: *hemisphere* (left, right); within-subject factor: *TMS* (OFA, Control)) was carried out separately for the performance accuracy of recalled job titles and names. Name retrieval performance of all four conditions (OFA/control TMS in the left/right hemisphere) was additionally tested against chance level by a one-sample *t* test. Reaction times of correct responses were analyzed in the same manner as the performance with a mixed ANOVA (between-subject factor: *hemisphere* (left, right); within-subject factor: *TMS* (OFA, Control)), separately for job titles and names. A Sidak corrected post hoc test was used for multiple comparisons (Field 2009).

## Results

### Job title retrieval

**Fig. 3:**
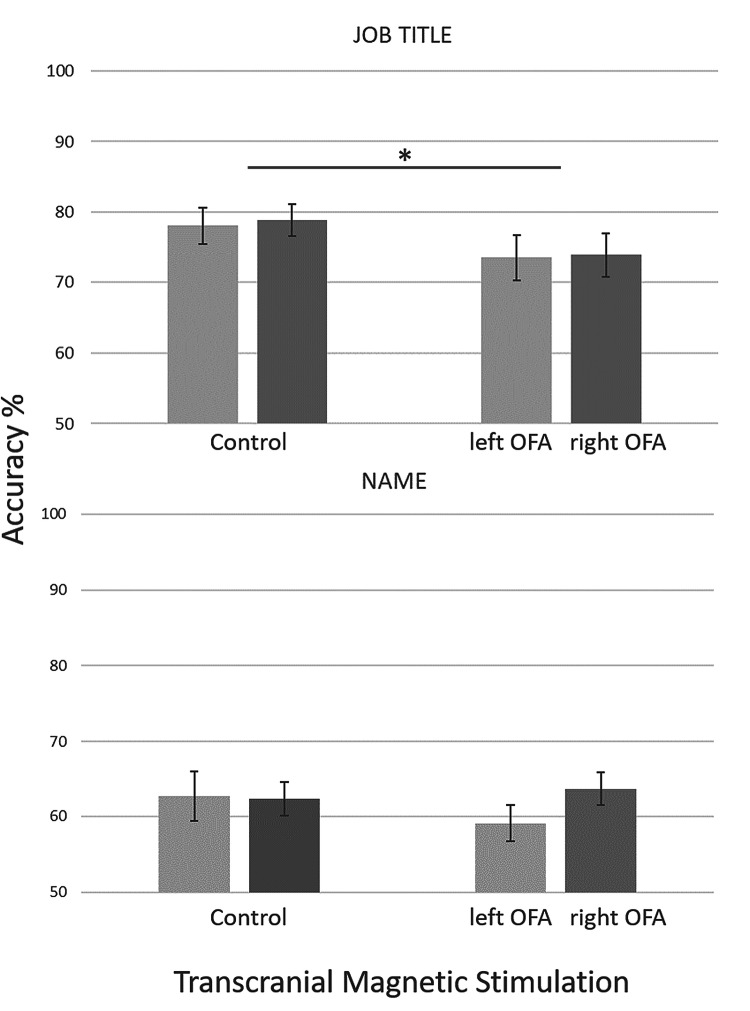
Performance for job title (up) and name retrieval (down) after OFA and control (Cz) stimulation, separately for left and right hemisphere-stimulated participants (mean ± SE; * ≤ 0.05)

TMS stimulation of the bilateral OFA during encoding reduced the proportion of correctly recalled job titles significantly (Fig. 3; main effect of *TMS:* (*F*(1,42) = 5.04, *p* = 0.030, *η*^2^_p_ = 0.107). This suggests that the OFA is important for the association of faces and related semantic information during face familiarization. The average performance as well as the effect of TMS was similar for the two participant groups with left or right OFA stimulation (main effect of *hemisphere* (*F*(1,42) = 0.03, *p* = 0.868, *η*^2^_p_ = 0.001; interaction of hemisphere and TMS: (*F*(1,42) = 0.13, *p* = 0.910, *η*^2^_p_ = 0.000), suggesting similar functions of the two hemispheres.

### Name retrieval

Unlike what we found for job title retrieval, TMS over the left or right OFA showed no influence on the correct retrieval of previously associated names (Fig. 3; main effect of TMS: F (1,42) = 0.40, *p* = 0.530, *η*^2^_p_ = 0.009). Note, however, that the performance for name retrieval was generally low [nonetheless, significantly different from chance level (*p* ≤ 0.001)], suggesting that the applied paradigm and the number of repetitions might not have been sufficient to create a proper face–name association. Neither the main effect of hemisphere, nor the hemisphere/TMS interaction was significant for the name retrieval performance [main effect of hemisphere: (*F*(1,42) = 0.46, *p* = 0.502, *η*^2^_p_ = 0.011; interaction: *F*(1,42) = 1.84, *p* = 0.182, *η*^2^_p_ = 0.042).

The reaction times of the correct responses showed neither a main effect of *TMS* (recall of job titles *F*(1,42) = 0.86, *p* = 0.358, *η*^2^_p_ = 0.020; recall of names: *F*(1,42) = 0.00, *p* = 0.949, *η*^2^_p_ = 0.000) nor of *hemisphere* (recall of job titles *F*(1,42) = 2.80, *p* = 0.102, *η*^2^_p_ = 0.063; recall of names: *F*(1,42) = 0.10, *p* = 0.748, *η*^2^_p_ = 0.002).

## Discussion

The major results of the current study can be summarized as follows. (1) Stimulating the OFA during the encoding of face-related semantic information reduces the correct recall of such associations for job titles, suggesting a causal role of the OFA in the association of familiar faces with related semantic information. (2) Contrary to prior findings, we did not observe hemispherical differences in this effect, suggesting a similar role of the left and right OFA in the formation of such associations.

The OFA is causally involved in semantic associations to familiar faces.

Experimental familiarization of unfamiliar faces can be performed by making fictional biographical, episodic, social or affective information available to the observers during an encoding or learning period. It is believed that rich experience with the identities facilitates the creation of a robust and long-lasting representation, modeling everyday familiarity better and facilitating the future identity recall of recognition memory. We applied TMS stimulation to the OFA during the encoding phase and this affected the subsequent recall of associated job titles with originally unfamiliar faces in both hemispheres negatively Therefore, it is likely that the OFA plays a role in the initial encoding of sensory and biographical information associations. Whether it also plays a role in other processes of recognition, such as information retrieval, is unclear and will require specifically targeting experiments. Nonetheless, our results suggest a causal involvement of OFA in the acquisition of identity-related semantic information. This conclusion is in line with prior studies showing that the OFA plays a causal role in the acquisition of face identity information as well as in the creation of cross-domain identity representation (Amado et al. 2018; Ambrus et al. 2019). Consequently, our results further support the idea that the OFA, presumably together with the other face-sensitive areas within the core network, plays a direct role in higher face-processing steps. This conclusion is consistent with prior studies showing that the OFA is involved in the formation of identity-specific memory traces as well as in face identity acquisition (). Therefore, it seems that the OFA is part of the network responsible for the identity-specific representation of a familiar face and that it also plays a role in the association of semantic information to the face of a person. Consequently, we suggest that the role of OFA and its interaction with downstream face-specific regions, such as the FFA and ATL, have to be re-evaluated in future studies. This conclusion is in line with previous lesion studies, which found normal FFA activations for faces in the absence of OFA (Rossion 2014), suggesting that the encoding of faces in the FFA is not directly dependent on its input from the OFA. This also argues against a strictly serial information processing in the core face network. Rather, it indicates that the OFA and FFA are either at similar hierarchical levels or that the FFA might even be activated independently of and earlier than the OFA (Jiang et al. 2011; Rossion 2014).

The reversed temporal course of activations is supported by BOLD latency mapping studies, which revealed somewhat earlier responses to faces in the FFA (2.02 s) than in the OFA (2.43 s; Jiang et al. 2011; Rossion et al. 2012). Note, however that there is no general agreement about this interpretation in the literature. For example, Yovel (2016) measured correlations between fMRI and EEG data and found an earlier activation onset in the OFA (104–124 ms) as compared to FFA (136–184 ms). However, this must not necessarily lead to the conclusion that the OFA is situated at a hierarchically lower processing level than the FFA. It rather suggests that the OFA is crucial to time-critical tasks, such as the discrimination of a familiar face from the face of a potentially dangerous person. Very early recognition of familiar faces is in line with recent EEG studies, which reported face identification already within 160 ms post-stimulus onset (Vida et al. 2017; Zimmermann et al. 2019). These findings, among others, contradict the simple hierarchical feedforward models of face perception (Atkinson and Adolphs 2011; Haxby et al. 2000), as these models consider OFA as the entry point of the network, responsible for the early perception of facial features (Guntupalli et al. 2017; Haxby et al. 2000).

Studies with patients having focal OFA lesions described an impairment in recognizing famous or personally familiar faces, but reported neither problems in object detection nor, more importantly, in face detection per se (Rossion 2014).These symptoms hint at the processing of general face detection in FFA and individual face recognition in OFA. On the other hand, the above-described lesion studies did not find face identity adaptation effects in the FFA (i.e., they found similar BOLD signals to identical and different faces). This suggests that the FFA nonetheless requires input from the OFA regarding the identity of the faces for successful individual face discrimination (Rossion 2014).

Altogether, it seems that the OFA, in addition to undeniably being involved in the low-level structural encoding of faces (Rotshtein et al. 2005), is likely to be involved in higher-level face-processing steps as well. This, in turn, casts doubt on the validity of the dominantly serial models of face processing (Bruce and Young 1986; Haxby et al. 2000) and suggests that the encoding behind the recognition of a familiar face is not limited to higher face-processing regions, but also includes the entire occipito-temporal network via recurrent feedback connections. Current models of face processing present a rather complex face-processing network, with multiple forward and feedback connections between each of its nodes (Duchaine and Yovel 2015). Image-independent face processing and semantic information are in close interplay across this network, explaining why semantic information is so effective when we get to know a previously unfamiliar person (Schwartz and Yovel 2016). However, in these models the OFA is still a part of the ‘early’ face-processing network and is involved in view-specific and part-based face processing (Duchaine and Yovel 2015).

The model of ‘non-hierarchical network of cortical face-selective areas’ of Rossion (2014), however, attributes the OFA a more sophisticated role than accepted by most. The author proposes a reformulated organization for early face recognition with input coming from early visual areas to the OFA, but also directly to the FFA, where face detection occurs. The connections between the FFA and the OFA have been so far always classified as feedback connections (Gschwind et al. 2012; Rossion et al. 2003). The reverse hierarchical neural model conversely describes an initial input from the FFA to the OFA, not via feedback, but rather via feedforward connections. This interpretation is supported by Kadosh et al. (2011), who report that the OFA receives information at a mid-latency processing stage, around 170 ms. Downstream processing steps refine the face representation further, through re-entrant feedback connections between higher-level areas, like the ATL, and ‘lower’-level visual areas. Indeed, Pyles et al. (2013) found strong connections between ATL and FFA as well as between the ATL and OFA. The densest connections were found, however, between the posterior and medial FFA and OFA. This reverse hierarchical neural model of individual face perception suggests the existence of a network with many re-entrant connections (for a detailed overview of person identification models, see Barton and Corrow (2016)).

Why the OFA is located at a more posterior position (compared to the FFA), while being involved in ‘higher’ face-processing steps than the FFA can be explained by the shared characteristics with the early, retinotopically organized occipital areas. Accordingly, the OFA shows robust position sensitivity (Guntupalli et al. 2017; Xu and Biederman 2010) as well as relatively small receptive fields (Grill‐Spector et al. 1998), and TMS over the right OFA impairs face shape discrimination (Freiwald et al. 2016). We know that differences in particular face features (such as the distance between eyes, mouth, nose, etc., or the size of facial parts) contribute to the recognition of individual faces. Hence, the posterior location of OFA is not due to its role in early and low-level face-processing steps, rather it suits best its complex function in recognizing facial identity. On the one hand, this hypothesis fits well with the study of Rotshtein et al. (2005), reporting that the inferior occipital gyrus (IOG) shows sensitivity to physical rather than to identity changes. On the other hand, it is also in line with studies showing face identity adaptation effects in OFA (Ewbank and Andrews 2008; Gauthier et al. 2000). Our current findings are in line with these studies, suggesting the causal role of the OFA in the association of semantic information to familiar faces.

### No effect of TMS on face-name associations

Although previous TMS studies reported the involvement of the OFA in the processing of face-name associations (Ambrus et al. 2019), the current experiments did not find such a relationship. The reason behind the different findings can be multifold. First, we found low performances for the name-face association task. This suggests that face-associated names are harder to remember than job titles (Burton et al. 2019). We can merely hypothesize that this is so because job titles are capable of eliciting visual associations with (stereo) typical features (for example when we hear the job title ‘cook’, we immediately imagine a person in a white coat, wearing a hat). Names, on the other hand, are more neutral and freer from such stereotypes (but not completely unaffected, see Zwebner et al., 2017). This makes it likely that the OFA, a dominantly visual region, might be less involved in the association of faces with names than with job titles. Future semantic-face associations studies therefore should adapt the number of training trials for names and job titles to obtain a similar baseline performance.

Second, it is also possible that jobs and names are associated with faces in different steps or even via different routes, and the TMS of OFA only affects the ones involved in encoding associations of a job to a face. However, virtually no study has tested this possibility with TMS so far. Tsukiura et al. (2010), using neuroimaging methods with a very similar paradigm to ours, found a greater BOLD signal in ATL for job title than for name retrievals. The fact that associated job titles are generally better recalled than associated names would also support a more serial model of person identification (Tsukiura et al. 2010) with the recall of face-related information occurring first, then being followed by the association of person-specific semantic information (such as job title) and finally by the association of the name of the person (Bruce and Young 1986).

### Hemispherical lateralization

We have not found significant differences in the association of semantic information to faces between the stimulation of the right and left OFA. This is at odds with prior studies showing robust hemispherical differences at the most anterior part of the temporal lobe, with the right hemisphere being more involved in the visual processing of familiar faces, and the left one being more involved in the association of faces and names (Chiou and Lambon Ralph 2018; Ranieri et al. 2015; Rice et al. 2018). This, together with our current results, suggests that the hemispherical differences of visual and semantic face-related information processing emerge at later steps, presumably between the OFA and the ATL.

### Limitation

One potential limitation of the current study is that the number of repetitions was not sufficient to successfully associate the names with the simultaneously seen faces. As the performance in the name recall task was low, we refrain from making conclusions regarding name-face associations. However, the fact that the job–face associations were affected by the TMS of both left and right OFAs suggests that the areas are indeed involved in the associations of person identity-related semantic information to faces.

Another general methodological constraint, which limits the interpretation of any TMS study, is that the stimulation effects may propagate from the target area towards other processing stages of the network as well (Ruff et al. 2009), limiting the causal interpretations.

### Summary

An identity learning task and the simultaneous disruption of face processing by TMS were used to test whether the OFA is involved in the association of semantic information with a face. The recall of face-associated job titles was reduced after stimulation of the OFA over either hemisphere. It suggests that the OFA participates in the processing, i.e., the encoding or learning of identity-related semantic information, suggesting its important role in face recognition. This, in turn, suggests, that the encoding that is part of the recognition of a familiar face is not limited to higher face-processing regions, but that the entire occipito-temporal network, including the OFA, is involved.

As mentioned above, we cannot exclude the possibility that the stimulation of the OFA may affect adjacent areas of the face network. What the outcome of our experiments indicates, however, is that the OFA is part of the network involved in higher face processing, presumably via recurrent feedback connection. Consequently, previous face-perception models, that attribute low-level processing to the OFA (Bruce and Young 1986; Haxby et al. 2000), require reconsideration. Overall, our results are more compatible with distributed and recurrent models of face perception (Barragan-Jason et al. 2013; Freiwald and Tsao 2010; Kravitz et al. 2013).
